# Neurotoxic Effect of Flavonol Myricetin in the Presence of Excess Copper

**DOI:** 10.3390/molecules26040845

**Published:** 2021-02-05

**Authors:** Anja Sadžak, Ignacija Vlašić, Zoran Kiralj, Marijana Batarelo, Nada Oršolić, Maja Jazvinšćak Jembrek, Ines Kušen, Suzana Šegota

**Affiliations:** 1 Division of Physical Chemistry, Ruđer Bošković Institute, 10000 Zagreb, Croatia; Anja.Sadzak@irb.hr; 2 Division of Molecular Medicine, Ruđer Bošković Institute, 10000 Zagreb, Croatia; Ignacija.Vlasic@irb.hr; 3 Department of Biology, Faculty of Science, University of Zagreb, 10000 Zagreb, Croatia; zkiralj.biol@pmf.hr (Z.K.); mbatarelo@stud.biol.pmf.hr (M.B.); norsolic@yahoo.com (N.O.); 4 Department of Psychology, Catholic University of Croatia, 10000 Zagreb, Croatia; 5 King’s College London, London WC2R 2LS, UK; ines.kusen@kcl.ac.uk

**Keywords:** myricetin, prooxidative activity, copper toxicity, atomic force microscopy, elasticity, roughness

## Abstract

Oxidative stress (OS) induced by the disturbed homeostasis of metal ions is one of the pivotal factors contributing to neurodegeneration. The aim of the present study was to investigate the effects of flavonoid myricetin on copper-induced toxicity in neuroblastoma SH-SY5Y cells. As determined by the MTT method, trypan blue exclusion assay and measurement of ATP production, myricetin heightened the toxic effects of copper and exacerbated cell death. It also increased copper-induced generation of reactive oxygen species, indicating the prooxidative nature of its action. Furthermore, myricetin provoked chromatin condensation and loss of membrane integrity without caspase-3 activation, suggesting the activation of both caspase-independent programmed cell death and necrosis. At the protein level, myricetin-induced upregulation of PARP-1 and decreased expression of Bcl-2, whereas copper-induced changes in the expression of p53, p73, Bax and NME1 were not further affected by myricetin. Inhibitors of ERK1/2 and JNK kinases, protein kinase A and L-type calcium channels exacerbated the toxic effects of myricetin, indicating the involvement of intracellular signaling pathways in cell death. We also employed atomic force microscopy (AFM) to evaluate the morphological and mechanical properties of SH-SY5Y cells at the nanoscale. Consistent with the cellular and molecular methods, this biophysical approach also revealed a myricetin-induced increase in cell surface roughness and reduced elasticity. Taken together, we demonstrated the adverse effects of myricetin, pointing out that caution is required when considering powerful antioxidants for adjuvant therapy in copper-related neurodegeneration.

## 1. Introduction

Neurodegenerative diseases, such as Alzheimer’s disease and Parkinson’s disease, are characterized by progressive synaptic and axonal degeneration that ultimately ends in neuronal death in specific regions of the brain. Oxidative stress (OS) is one of the major pathological mechanisms contributing to neuronal damage and loss of function in these devastating diseases. OS is generated when the balance between production and elimination of free radicals is disturbed. Free radicals, which include a broad-spectrum of reactive oxygen and nitrogen species (ROS and RNS, respectively), are highly reactive moieties with an unpaired electron that are capable of inducing oxidative damage of cellular macromolecules and other components, resulting in their functional impairment [1,2].

One of the pivotal factors contributing to OS conditions in neurodegenerative disorders is related to the systemic imbalance of copper and other redox-active transition metals [3,4,5]. Despite some inconsistencies in the literature, it seems that levels of copper are elevated in the serum of patients suffering from Alzheimer’s disease [6]. Copper is essential for the catalytic activity of many brain proteins involved in basic neuronal functions, such as respiration, antioxidative defense, myelin formation and catecholamine synthesis. When present in excess, copper ions initiate redox cycling reactions and ROS formation, creating an oxidative environment that ultimately results in neuronal injury [7,8]. A complex system of copper transporters and chaperones has been evolved to deliver copper to copper-containing enzymes while preventing the cellular accumulation of free copper and OS induction [9]. The prooxidative effects of copper are mostly attributed to Cu^+^ ions (cuprous ions, reduced state), which catalyze the formation of extremely toxic hydroxyl radicals through the decomposition of hydrogen peroxide in a Fenton-type reaction. In a reaction with molecular oxygen, Cu^+^ ions yield superoxide anions and regenerate Cu^2+^ ions, which are thus ready for a new cycle of ROS formation. Hydroxyl radicals are the most powerful oxidizing species that may induce lipid peroxidation and oxidative damage of all biological targets. Superoxide anions, although less reactive, may also contribute to oxidative damage if not removed by the superoxide dismutase [7,10]. In addition, when the intracellular mechanisms involved in copper coordination are saturated, free copper ions may exert deleterious effects through direct and nonspecific binding to sulfhydryl groups (thiols) and amino groups of various cellular proteins, thus disturbing their normal cellular functions. Copper can also interact with bases in nucleic acids and induce their conformational changes and oxidative damage [10], as well as catalyze autooxidation of carbohydrates and unsaturated lipids [11]. Furthermore, copper overload is able to disturb regular copper/protein interactions and promote the self-aggregation ability of specific proteins involved in neurodegeneration [4]. Copper attachment to cellular proteins and changes in protein folding has been suggested as one of the main contributing factors to copper-mediated death events [12]. Finally, it is known that increased copper may compromise the proteolytic activity of the ubiquitin-proteasome system, further promoting neuronal dysfunction in neurodegeneration [13].

Despite significant efforts, current therapeutic options for neurodegenerative diseases are limited and aim to treat only the symptoms. In both the scientific community and the general population, there is a great interest in compounds of natural origin that could potentially be effective against pathological processes and the progression of diseases. In that regard, flavonoids have attracted much attention as a promising therapeutic approach against oxidative injury. They may achieve antioxidative effects by direct ROS scavenging, via stimulation of endogenous antioxidative mechanisms and through metal chelation. Additionally, they act as modifiers of ROS-induced signaling and may attenuate neuronal death by preventing downstream signal transduction [5,14,15,16]. However, besides these desirable effects, flavonoids may exert pro-oxidant activity, particularly in the presence of metal ions [17,18,19].

Myricetin is a flavonoid from the flavonol group commonly found in various berries, vegetables, and beverages. Its skeleton is composed of two aromatic rings, termed A and B, which are bridged by a heterocyclic pyran ring C [18,20]. The neuroprotective effects of myricetin have been reported in several studies and are largely attributed to antioxidative and anti-inflammatory effects [21,22]. Powerful antioxidative activity is based on the polyphenolic structure enriched in numerous hydroxyl (OH) groups. Besides ROS trapping abilities, myricetin also possesses metal chelation properties [20,23]. In primary cultures of cortical neurons, myricetin attenuated glutamate-mediated toxicity by reducing intracellular Ca^2+^ overload, glutamate-provoked increases in ROS production and caspase-3 activation [24]. Regarding Alzheimer’s disease, it prevented amyloid β (Aβ)-induced toxicity and Aβ aggregation [25].

Interactions between myricetin and copper have not yet been studied in neuronal cells. Considering that the presence of copper ions can be important for the switch from antioxidant to pro-oxidant behavior of flavonoids, the aim of the present study was to investigate the effects of myricetin in copper-induced OS conditions and to reveal the cellular events and molecular mechanisms that underlie its effects. The study was performed in SH-SY5Y neuroblastoma cell line that is routinely used for studying various aspects of neuronal biology, including the cellular response to OS-induced injury [26]. In addition to standard methods of cellular and molecular biology, we employed atomic force microscopy (AFM) as an advanced biophysical tool to better reveal the subtle structural and mechanical changes resulting from cell exposure to OS. AFM was used for quantitative imaging of the cell surface and membrane topography, whereas its non-imaging mode provided nanomechanical properties usually reported as cell elasticity. Both the imaging and non-imaging AFM modes can elucidate valuable information about cell membranes and cytoskeleton architecture, which can both be affected by environmental conditions, including the oxidative insult [27,28].

## 2. Results

### 2.1. Treatment with Myricetin Exacerbated Copper-Induced Decrease of Viability in SH-SY5Y Cells

**Figure 1:**
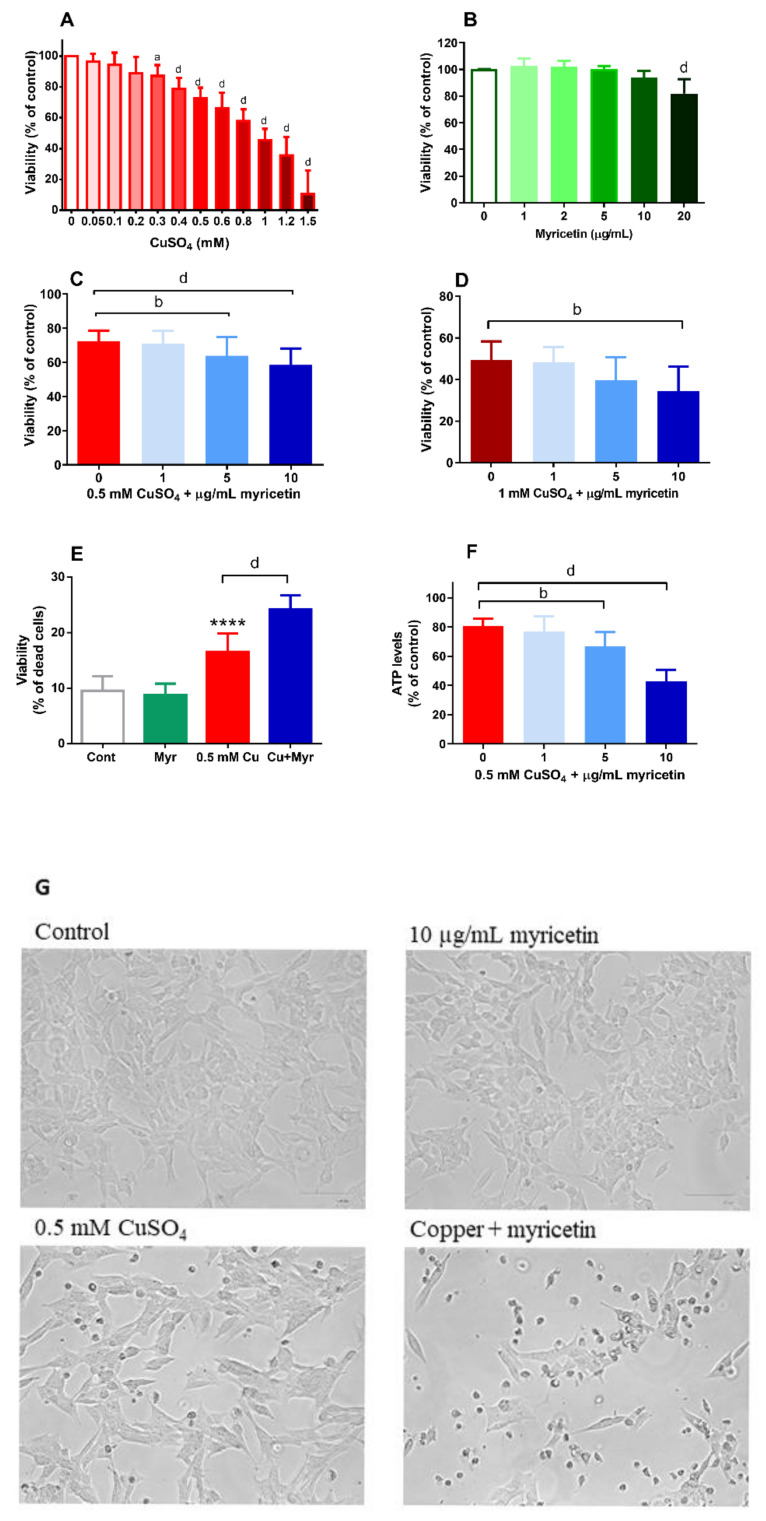
Neurotoxic effect of myricetin in SH-SY5Y cells exposed to excess copper. SH-SY5Y cells were incubated with increasing concentrations of CuSO_4_ (**A**) and myricetin (**B**) for 24 h, and their viability was assessed by 3-(4,5-dimethylthiazol-2yl)2,5-dyphenyl-2*H*-tetrazolium bromide (MTT) assay. MTT also revealed that in a dose-dependent manner, myricetin promoted mild (**C**) and more severe (**D**) copper-induced decrease in viability. The detrimental effect of myricetin in the presence of 0.5 mM CuSO_4_ was further confirmed by trypan blue exclusion assay (**E**) and determination of ATP content (**F**). All the measurements were performed 24 h from the beginning of treatment. Statistical analysis of the obtained data was performed by one-way ANOVA followed by Dunnett’s (**A** and **B**) or Tukey’s (**C**–**F**) multiple comparison tests. **** *p* < 0.0001 in comparison to control group; ^a^ *p* < 0.05, ^b^ *p* < 0.01, ^c^ *p* < 0.001, and ^d^ *p* < 0.0001 vs. 0 group (either control or 0.5 mM copper). Data are expressed as means ± SD from 6 to 8 independent experiments performed in quadruplets for MTT assay, 5 independent experiments performed in duplicates for trypan blue exclusion method and 4 independent experiments performed in triplicates for the determination of ATP content. The morphological appearance of treated cells (**G**) is also presented.

As shown in Figure 1A, exposure to copper (0.1–1.5 mM) for 24 h reduced viability of neuroblastoma SH-SY5Y cells in a dose-dependent manner. For further studies, we selected a 0.5 mM concentration of CuSO_4_ as this concentration consistently induced significant, but neither too small nor too extensive cell death. Flavonoid myricetin was also capable of reducing the viability of SH-SY5Y cells when applied at a concentration of 20 µg/mL (Figure 1B). Hence, for further studies, we used concentrations of myricetin up to 10 µg/mL (31.4 µM). When applied together with 0.5 mM copper, myricetin at 5 and 10 µg/mL exacerbated the toxic effects of copper and further reduced viability of SH-SY5Y cells. As determined by the MTT assay, in the presence of both copper and myricetin, survival was 58% (58.49 ± 9.61), whereas, in cells exposed to 0.5 mM CuSO_4_ alone, viability was 73% (72.53 ± 6.11) (Figure 1C). The neurotoxic effect of myricetin was also evident in the presence of 1 mM CuSO_4_ (Figure 1D). At this concentration of copper, myricetin applied at 10 µg/mL concentration decreased viability from 49% to 35%. This pro-death effect of myricetin was also confirmed by the trypan blue method (Figure 1E). Finally, we monitored ATP levels, as they indicate the overall metabolic status and viability. Consistently with our other results, copper applied alone reduced ATP content, and this decrease was further promoted in the presence of 10 µg/mL myricetin. Following exposure to copper and myricetin, the ATP content was depleted to 43% of the basal value detected in control cells (Figure 1F). The morphological appearance of treated cells is represented in Figure 1G. Similar to the control group, SH-SY5Y cells treated with 10 µg/mL myricetin showed an epithelial-like flat phenotype. In the copper-treated group, the number of round cells detached from the surface was slightly increased. The volume of attached cells looked increased, and boundaries between cells were not so obvious. Visual inspection under the microscope also suggested a reduced cell density in comparison to the control and myricetin-treated group. The addition of myricetin further increased the number of dead cells floating in the medium. A proportion of cells exhibited rounded morphology and reduced volume while remaining attached to the plastic surface.

### 2.2. Effects of Myricetin on ROS Generation and GSH Content in the Presence of Excess Copper

**Figure 2:**
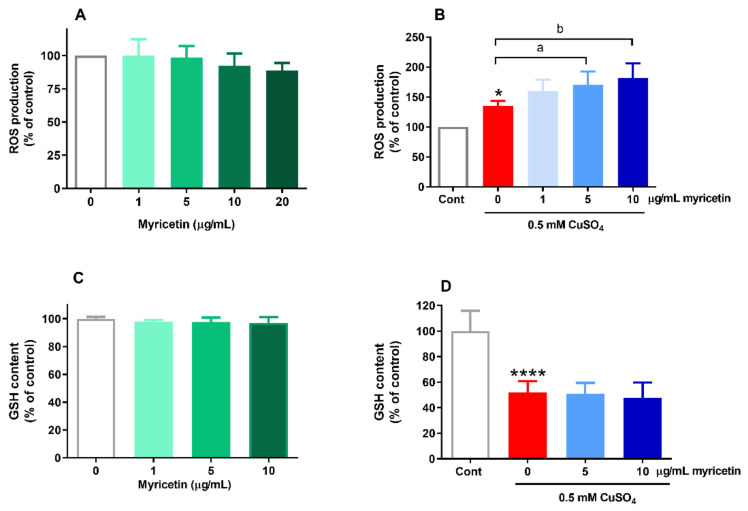
Effects of myricetin on reactive oxygen species (ROS) production and glutathione (GSH) content in copper overload. SH-SY5Y cells were incubated with different concentrations of myricetin (1–20 µg/mL) in the absence or presence of 0.5 mM CuSO_4_ for 24 h. ROS levels were determined by measuring fluorescence intensity after incubation with 2’,7’-dichlorofluorescin diacetate (DCF-DA). Myricetin applied alone did not affect intracellular ROS generation up to a concentration of 20 µg/mL (**A**). Exposure to 0.5 mM CuSO_4_ increased the production of ROS, and this effect was further promoted by 5 and 10 µg/mL myricetin (**B**). Like ROS production, GSH content was not changed in SH-SY5Y cells exposed only to 1–10 µg/mL myricetin (**C**). Copper ions applied alone depleted the intracellular amount of GSH, and concomitant treatment with myricetin did not induce further changes (**D**). Data represent the mean ± SD from four independent experiments performed in quadruplets (ROS) or triplicates (GSH). **** *p* < 0.0001 vs. control (one-way ANOVA followed by post hoc Dunnett’s test); ^a^ *p* < 0.05, ^b^ *p* < 0.01 vs. copper-treated group (one-way ANOVA followed by post hoc Tukey’s test).

Based on reports that indicated both antioxidative and prooxidative effects of myricetin, we next examined if the neurotoxic effects of myricetin were associated with changes in ROS production and GSH content. As shown in Figure 2A, myricetin applied alone did not modify the production of ROS, although a tendency towards a reduction in ROS content was evident for 10 and 20 µg/mL myricetin. Copper at 0.5 mM concentration induced an increase in ROS production, which was further augmented in the presence of 5 and 10 µg/mL myricetin. In copper-treated cells, ROS levels were elevated by 35%, whereas in the presence of 5 and 10 µg/mL of myricetin, ROS production was increased by 70% and 82%, respectively, in comparison to the control group (Figure 2B).

Increased ROS generation may affect stores of intracellular antioxidants. As GSH is the major molecule of nonenzymatic antioxidant defense, we looked for the eventual changes in GSH content. We failed to find alterations in GSH amount when SH-SY5Y cells were treated with myricetin only (Figure 2C). In cells that were exposed to 0.5 mM CuSO_4_, GSH content was depleted to 52% of the basal value. No further reduction of GSH was detected in the presence of 5 or 10 µg/mL myricetin (GSH levels were 51% and 48%, respectively, of the values detected in control SH-SY5Y cells; Figure 2D).

### 2.3. Myricetin Promoted Copper-Induced Changes in Chromatin Condensation and Stimulated LDH Release

Depending on the severity of the oxidative injury, ROS-initiated cell death can be broadly classified as apoptotic or necrotic. During apoptosis, also termed as programmed cell death, an increased generation of ROS may trigger caspase-dependent or caspase-independent neuronal death that are both characterized by chromatin condensation. On the other hand, chromatin condensation and preservation of cell membrane integrity may help in differentiating apoptosis from necrosis. Hence, to distinguish between these possibilities, we monitored caspase-3/7 activity, leakage of cytoplasmic enzyme lactate dehydrogenase (LDH) into the surrounding medium as an indicator of cell membrane integrity, and chromatin density.

**Figure 3:**
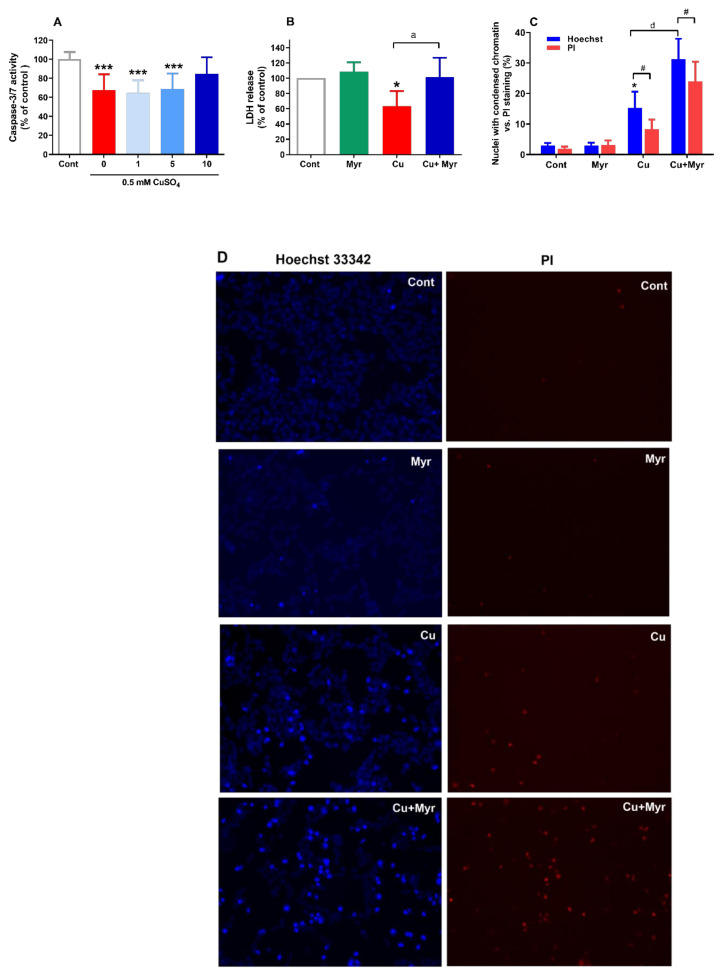
Cell death analysis by measuring caspase-3/7 activity, LDH leakage and nuclear staining with Hoechst 33342 and PI. At 24 h from the beginning of the treatment with copper and myricetin, activation of executioner caspases-3/7 was measured using the caspase-Glo 3/7 assay system (**A**). Exposure to copper, alone or in the presence of 1 and 5 µg/mL myricetin reduced caspase 3/7 activity. LDH activity in cells exposed to Cu^2+^ ions was below values observed in control cells. Concomitant treatment with 10 µg/mL myricetin increased LDH activity, thus reflecting a negative effect on the cell membrane integrity (**B**). Morphological appearance of chromatin was monitored by staining with nuclear dye Hoechst 33342, whereas the proportion of cells in late apoptosis and necrosis was determined by PI. Exposure to 0.5 mM CuSO_4_ induced nuclear, as well as late apoptotic/necrotic changes. In the presence of 10 µg/mL myricetin, these changes were further exacerbated (**C**). Representative photographs after staining with Hoechst 33342 and PI are represented in (**D**) (* *p* < 0.05, *** *p* < 0.001 vs. control; ^a^ *p* < 0.05, ^d^ *p* < 0.0001 vs. copper-treated group one-way ANOVA and post hoc Tukey’s test; ^#^ *p* < 0.05, paired *t*-test).

At the 24 h time point, caspase-3/7 activity was reduced by 32% in SH-SY5Y cells treated with copper. This reduction was also evident in cells exposed to copper and 1 and 5 µg/mL myricetin (35% and 31%, respectively). The observed decrease in activity was significant in comparison to the control group, whereas other changes between groups were not found with Tukey’s test following one-way ANOVA (Figure 3A). As a positive control for caspase-3/7 activation, SH-SY5Y cells were treated with staurosporine, a well-known inducer of apoptosis via the intrinsic apoptotic pathway. Applied at 100 nM concentration, staurosporine reduced viability of SH-SY5Y cells by 41% and induced an almost 5-fold increase in caspase-3/7 activity (data not shown).

We next examined leakage of the cytoplasmic enzyme LDH into the culturing medium. LDH is released from cells with compromised membrane integrity. As represented in Figure 3B, exposure to myricetin alone did not modify membrane permeability. In cells exposed solely to copper, LDH activity was below the basal values observed in the control group due to the LDH inactivation in the presence of Cu^2+^ ions [29]. However, when SH-SY5Y cells were treated with both copper and myricetin, a pronounced increase of LDH activity (60%) was observed, indicating that exposure to myricetin significantly affected the maintenance of membrane integrity.

Effects of different treatments on chromatin morphology were assessed by staining nuclei with the chromatin dye Hoechst 33342 (Figure 3C,D). In the presence of 0.5 mM CuSO_4_, the number of cells with condensed chromatin was increased from 2.9% to 15.3% (*p* < 0.05). The addition of myricetin doubled the number of cells exhibiting bright nuclear condensation (31.0%), thus indicating the proapoptotic effect of myricetin in a copper-enriched environment. We simultaneously stained nuclei with propidium iodide (PI), a nuclear dye that labels DNA of late apoptotic and necrotic cells. Similar to Hoechst staining, treatment with copper increased the number of PI-positive cells from 1.9% to 8.4%, and this number was further increased to 23.9% in the presence of myricetin. In both the copper and copper + myricetin groups, the number of nuclei exhibiting bright staining exceeded the number of PI-positive cells, but the effect was more pronounced in cells exposed solely to copper.

### 2.4. Effects of Copper and Myricetin on the Expression of Proteins Involved in Oxidative Response and Cell Death

**Figure 4:**
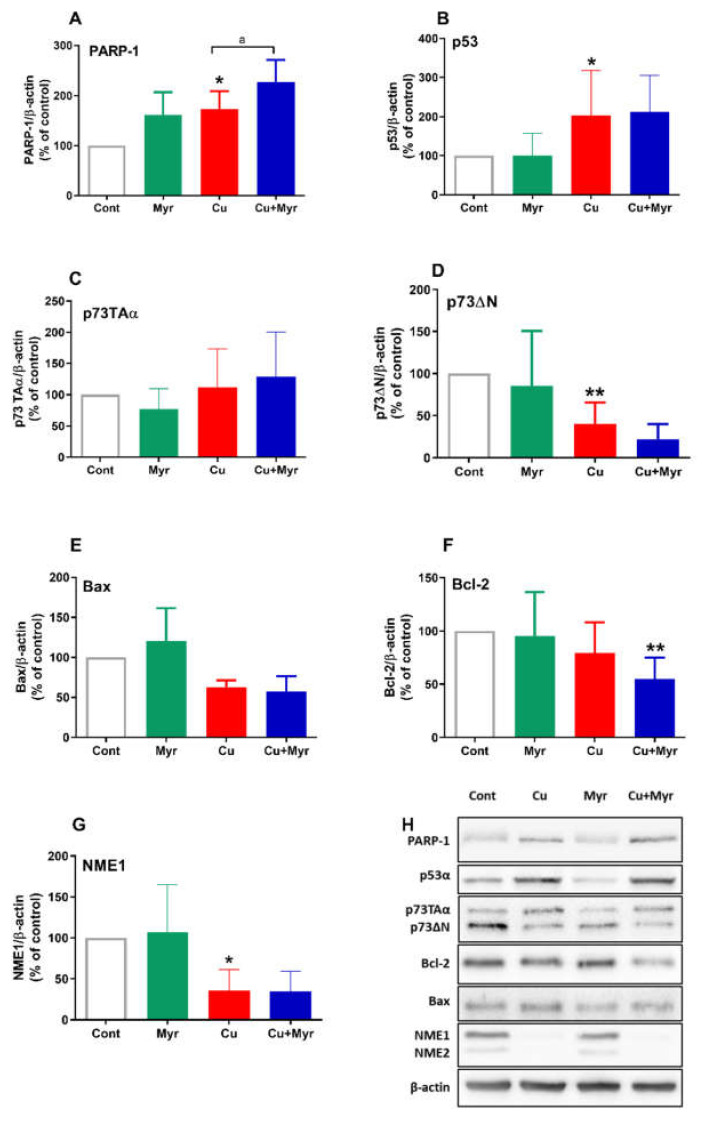
Myricetin-induced changes in the expression of apoptotic and oxidative stress-related proteins. Effects on the expression of PARP-1 (**A**), p53 (**B**), TAp73α (**C**), ΔNp73α (**D**), Bax (**E**), Bcl2 (**F**) and NME1/NME2 (**G**) were monitored 24 h from the beginning of treatment with 0.5 mM CuSO_4_ and 10 µg/mL myricetin. Proteins of total cell extracts were separated by polyacrylamide gel electrophoresis and transferred to nitrocellulose membranes. Blots were probed with primary antibodies, followed by the appropriate horseradish peroxidase-labeled secondary antibodies. Immunoreactivity was detected using enhanced chemiluminescence. β-actin was used as a loading control for normalization. Data are expressed as mean ± SD from 3 to 5 independently prepared cell lysates. Exposure to copper upregulated expression of poly(ADP-ribose)polymerase-1 (PARP)-1 and the observed effect was further increased by myricetin treatment. Copper also elevated levels of p53 and depleted levels of p73ΔN and NME1, but these changes were not further modified by myricetin. Expression of Bax and p73Taα was not affected by either treatment. Combined treatment with copper and myricetin induced a decrease in the expression of Bcl-2. Following densitometric analysis, obtained data were analyzed with one-way ANOVA followed by Tukey’s test (* *p* < 0.05, ** *p* < 0.01 vs. control; ^a^ *p* < 0.05 in comparison to the copper-treated group). Representative Western blots are also presented (**H**).

To better elucidate the molecular mechanisms of the neurotoxic effects of copper and myricetin, we looked for changes in the expression of proteins involved in the OS response and regulation of death pathways. Poly(ADP-ribose) polymerase 1 (PARP-1) is a nuclear enzyme that acts as a sensor of DNA damage and has an important role in the regulation of DNA repair. Its expression was increased by 74% in copper-treated SH-SY5Y cells (*p* = 0.0327, one-way ANOVA followed by Tukey’s test). When applied together with copper, myricetin induced an additional increase in PARP-1 expression (also *p* = 0.0327), thus reflecting more pronounced DNA damage during their combined treatment (Figure 4A). Quantitatively, PARP-1 expression was upregulated by 127% in comparison to the control, i.e., by a further 73% if compared to copper. Interestingly, myricetin applied alone upregulated the expression of PARP-1 by 61%, although this effect did not reach statistical significance (*p* = 0.1209). The transcription factor p53 is another cellular sensor of DNA damage. Its transcriptional activity may play a prominent role in pro-death events during oxidative injury [30]. Following copper treatment, protein levels of p53 were increased by 103%, but no further changes were induced by myricetin (Figure 4B). We also analyzed the expression of two p73 isoforms: TAp73α (full length) and ΔNp73α (truncated at the N terminus). As represented in Figure 4C,D, treatments with copper and/or myricetin did not modify levels of the TAp73α isoform, whereas levels of ΔNp73α were depleted by 60% following treatment with copper (*p* = 0.0014). During combined treatment, a trend towards further reduction of expression was visible (45% in comparison with copper-treated cells), although without statistical significance (*p* = 0.6371).

The Bcl-2 family of pro- and antiapoptotic proteins can be involved in the regulation of cell survival or death in many cell types, including neurons. The Bax protein is a transcriptional target of p53 involved in membrane permeabilization and the release of proapoptotic effectors, but its expression was neither affected by copper nor myricetin exposure (Figure 4E). By regulating the release of death-promoting factors, Bcl-2, an antiapoptotic member of the Bcl-2 family, confers resistance to cell death. Its expression was downregulated by 45% (*p* = 0.0226) in cells treated with copper and myricetin, suggesting that Bcl-2 is involved in the neurotoxic effects of myricetin (Figure 4F).

Finally, we monitored changes in the expression of the nucleoside diphosphate kinase NME1 that may also participate in the regulation of transcription and DNA repair [31]. Expression of the NME1 protein was downregulated by 64% and 66% in SH-SY5Y cells treated with copper or copper and myricetin, respectively (Figure 4G). A similar trend of changes was also observed for the NME2 protein (data not shown).

### 2.5. Effects of the Inhibitors of OS-Related Signaling Pathways on Copper and Myricetin-Induced Neuronal Death

**Figure 5:**
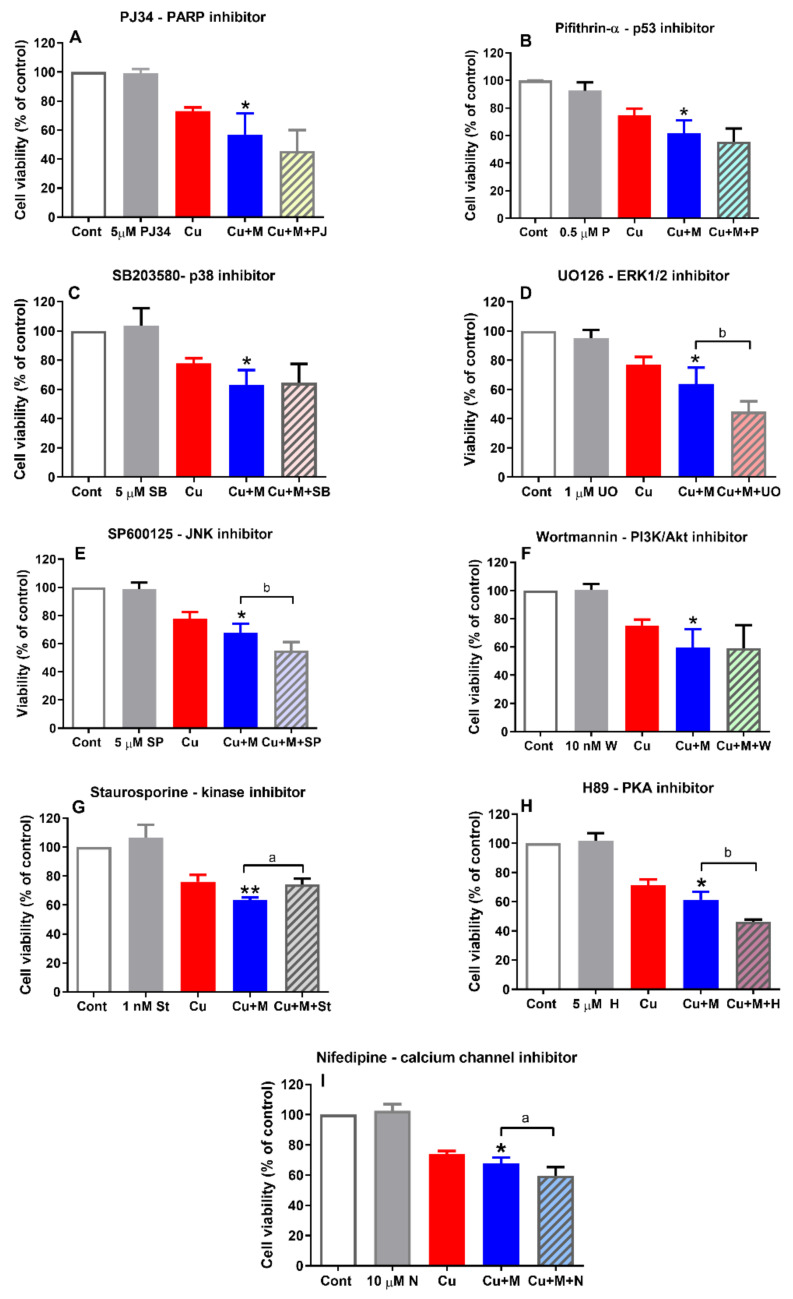
Effects of various inhibitors of oxidative stress (OS)-related proteins and signaling pathways on the neurotoxic effect of myricetin. SH-SY5Y cells were treated with the following inhibitors: PJ34-PARP inhibitor (**A**), pifithrin-α-p53 inhibitor (**B**), SB203580-p38 inhibitor (**C**), UO126-extracellular signal-regulated kinases 1/2 (ERK1/2) inhibitor (**D**), SP600125-c-Jun N-terminal kinase (JNK) inhibitor (**E**), wortmannin-phosphatidylinositol-3-kinase (PI3K)/protein kinase B (Akt) inhibitor (**F**), staurosporine-nonspecific kinase inhibitor (**G**), H-89-PKA inhibitor (**H**) and nifedipine-calcium channel inhibitor (**I**). Inhibitors were applied 1 h prior to and during the 24 h treatment with copper and myricetin. Inhibitors of PARP-1, ERK1/2, JNK, protein kinase (PKA) and voltage-gated calcium channels promoted the toxic effects of myricetin, further reducing the survival of SH-SY5Y cells. Staurosporine prevented the toxic effects of myricetin, whereas inhibitors of p53, p38 and PI3K/Akt pathways were without effect. Values are expressed as means ± SD from 4 to 7 independent experiments performed in quadruplets. * *p* < 0.05, ** *p* < 0.01 vs. 0.5 mM CuSO_4_ alone, ^a^ *p* < 0.05, ^b^ *p* < 0.01 vs. copper + myricetin treatment (one-way ANOVA followed by post hoc Tukey’s test)**.**

We used a pharmacological approach to further investigate the role of OS-related proteins and signaling pathways on the neurotoxic effect of myricetin. In the presence of the PARP-1 inhibitor VIII (PJ34), the toxic effect of myricetin was more pronounced (Figure 5A). In contrast, pifithrin-α, an inhibitor of transcriptional p53 activity, did not modulate the neurotoxic effect of myricetin in copper-induced OS conditions (Figure 5B). As p38/p53 signaling may contribute to the death outcome in OS, we examined the effect of SB203580, a p38 inhibitor, on the viability of SH-SY5Y cells concomitantly treated with copper and myricetin. Similar to pifithrin-α, SB203580 did not affect the toxic effect of myricetin (Figure 5C). We also monitored the effects of inhibitors of extracellular signal-regulated kinases 1/2 (ERK1/2) and c-Jun N-terminal kinase (JNK). Together with p38, kinases JNK and ERK1/2 belong to mitogen-activated protein kinases (MAPKs) that are commonly involved in the cellular response to environmental stressors and initiation of death events. UO126 is a highly selective inhibitor of the mitogen-activated protein kinase MEK1/2 that phosphorylates and activates kinases ERK1/2, whereas SP600125 acts as a JNK inhibitor. As represented in Figure 5D,E, both inhibitors promoted toxic effects of myricetin and additionally reduced the survival of SH-SY5Y cells. As the neurotoxic effects of drugs could be mediated through the inhibition of the PI3K/Akt signaling pathway, we applied wortmannin, a specific inhibitor of PI3K, which is an Akt activator. However, the effect of myricetin was not affected when cells were concomitantly treated with copper, myricetin and wortmannin (Figure 5F). Among all the tested inhibitors, only staurosporine, a broad-spectrum kinase inhibitor, was capable of preventing the neurotoxic effect of myricetin (Figure 5G). H89, a protein kinase (PKA) inhibitor, exacerbated the toxic effects of myricetin and reduced viability of SH-SY5Y cells (Figure 5H). Finally, as Ca^2+^ ions could have an important role in the initiation of a death cascade—and considering that changes in intracellular calcium levels could be mediated by L-type voltage-gated calcium channels (VGCC)—we used nifedipine, a drug that blocks calcium entry through the L-type VGCC. As shown in Figure 5G, a non-toxic concentration of nifedipine potentiated the toxic effects of myricetin.

### 2.6. Effects of Myricetin and Copper on the Morphological Properties of SH-SY5Y Cells at the Nanoscale

AFM imaging was performed to obtain detailed information about the morphological changes of neuroblastoma SH-SY5Y cells upon treatment with 10 μg/mL myricetin, 0.5 mM CuSO_4_, and both myricetin and copper. Besides height images with high spatial resolutions, detailed information (cross-section profiles, roughness parameters, namely *Z* range, *R*_q_ and *R*_a_) about the induced structural changes during the treatments were extracted. *Z* range is the maximal distance between the highest and the deepest point on the scanned surface area, *R*_a_ is the average roughness, and *R*_q_ denotes the root mean square roughness.

**Figure 6:**
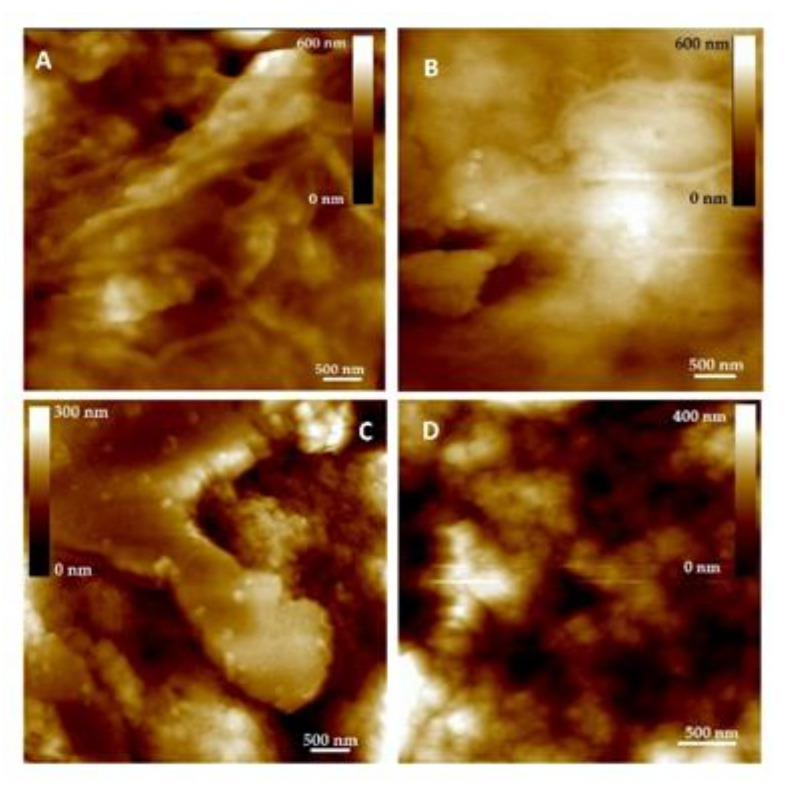
2D-height images of neuroblastoma SH-SY5Y cells using AFM: (**A**) control cell; (**B**) cell treated with 10 μg/mL myricetin for 24 h; (**C**) cell treated with copper (0.5 mM CuSO_4_) for 24 h; (**D**) cell treated with myricetin and copper (same concentrations as in the individual treatment) for 24 h. Scan area 5 μm × 5 μm and vertical scales are denoted on the images.

**Figure 7:**
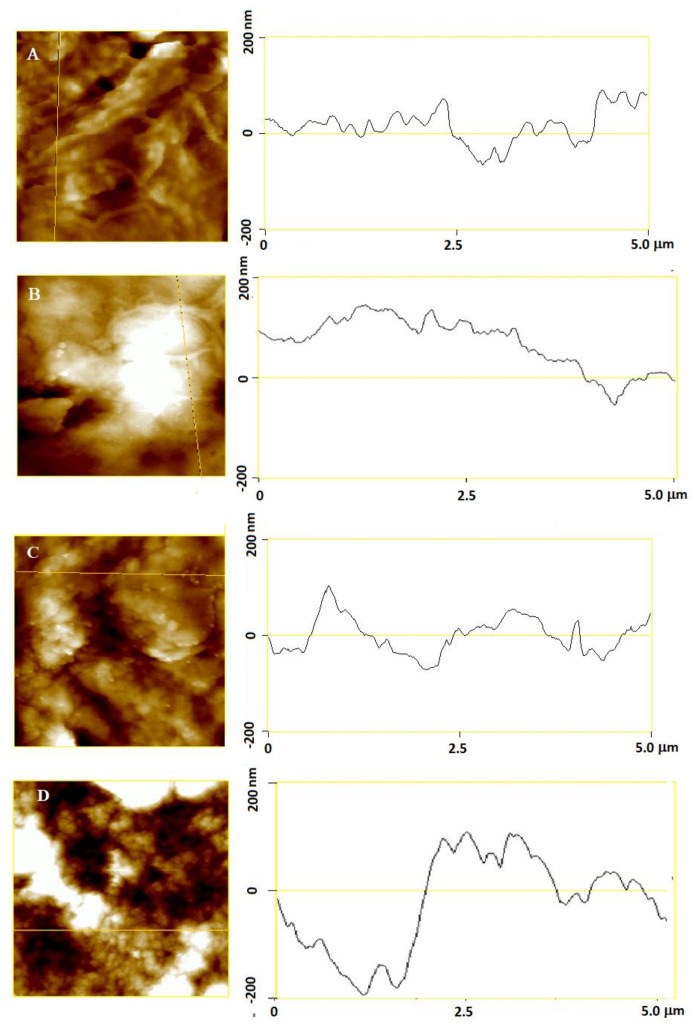
2D-height images (left) and height section profiles along the yellow line on the height image (right) of neuroblastoma SH-SY5Y cells using atomic force microscopy (AFM): (**A**) control cell without treatment; (**B**) cell treated with 10 μg/mL myricetin for 24 h; (**C**) cell treated with copper (0.5 mM CuSO_4_) for 24 h; (**D**) cell treated with myricetin and copper (same concentrations as in the individual treatment) for 24 h. The Y-scale on cross-section profiles is the same in all images and amounts to 400 nm.

**Figure 8:**
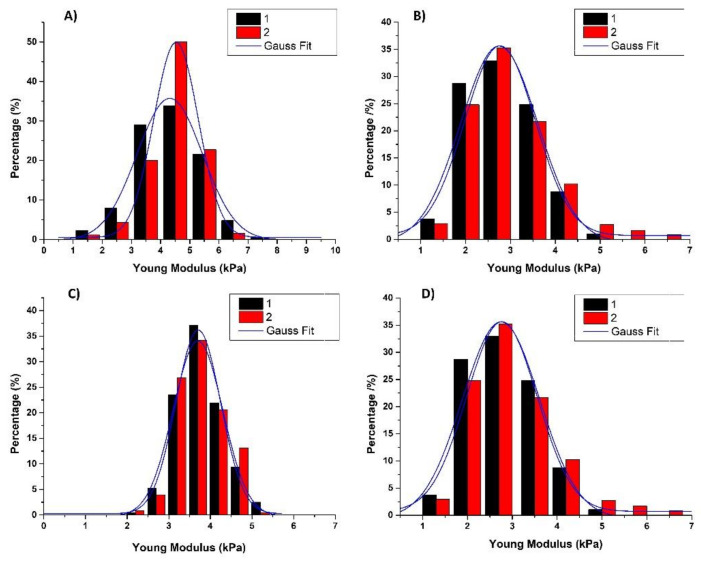
Elasticity histograms of neuroblastoma SH-SY5Y cells from two independent measurements using force spectroscopy: (**A**) control cells, (*N*_cell_ = 8), *N*_fc_ = 440; (**B**) cell treated with 10 μg/mL myricetin for 24 h, (*N*_cell_ = 8), *N*_fc_ = 480; (**C**) cells treated with copper (0.5 mM CuSO_4_) for 24 h, (*N*_cell_ = 8), *N*_fc_ = 480; (**D**) cells treated with myricetin and copper for 24 h, (*N*_cell_ = 8), *N*_fc_ = 480. Histograms were fitted with the Gauss function, indicated by blue lines.

Relatively clear and distinct protrusions were found on the control cells, indicating a presence of organized filament structures (Figure 6A) appearing up to 100 nm in height (Figure 7A). Even intertwined, filamentous structures that are clearly separated from each other are distributed homogeneously throughout the scanning area, forming cavities in the interspace between themselves. After myricetin treatment, the filamentous structures were still discernible though the cavity between them was smaller (see Figure 7B), which is reflected in a smoother cell surface structure compared to the untreated cells (Figure 8A,B for control cell and myricetin-treated cell, respectively). During treatment with copper, the filamentous structures could no longer be distinguished and observed (Figure 6C), and only aggregate-like structures appeared. These aggregate-like structures partially (45%) covered the cell surface, indicating that the aggregation process was heightened, while in SH-SY5Y cells simultaneously treated with myricetin and copper (Figure 6D), almost the whole surface (85%) was covered with aggregates. The visible cell surface covered by aggregates was no longer smooth, as was the case of those treated with copper, but took on the shape of two-dimensionally strung tooth-like structures with a height of up to 300 nm (Figure 7D).

To obtain additional data on the morphology of neuroblastoma SH-SY5Y cells, the surface roughness of treated cells was calculated by selecting eight regions of 25 μm^2^ from the AFM height images (Table 1). Representative AFM height images and the corresponding cross-section height profiles (Figure 7) indicate that the surface roughness of the cells changed during the different treatments (Table 1). In comparison to the control cell, slight differences on the surface of the neuroblastoma cells treated with 10 μg/mL myricetin were indicated by higher *Z* range and *R*_a_ values (10% and 6%, respectively). In contrast, when compared to the control cells, the surface of the copper-treated cells was significantly rougher, and the formation of aggregate-like structures on the cell surface was reflected in higher *Z* range, *R*_q_ and *R*_a_ values, increased by 46%, 40% and 64%, respectively, indicating the prominent growth of formed aggregate–like structures. Interestingly, *R*_a_ and *R*_q_ values of the myricetin and copper-treated neuroblastoma cells were further increased by 31% and 34%, respectively (Figure 7) in comparison to the copper-treated cells, indicating the stronger effect of myricetin in the presence of copper on the morphology of the neuroblastoma SH-SY5Y cells.

### 2.7. Effects of Myricetin and Copper on the Nanomechanical Properties of Neuroblastoma SH-SY5Y Cells

**Figure 9:**
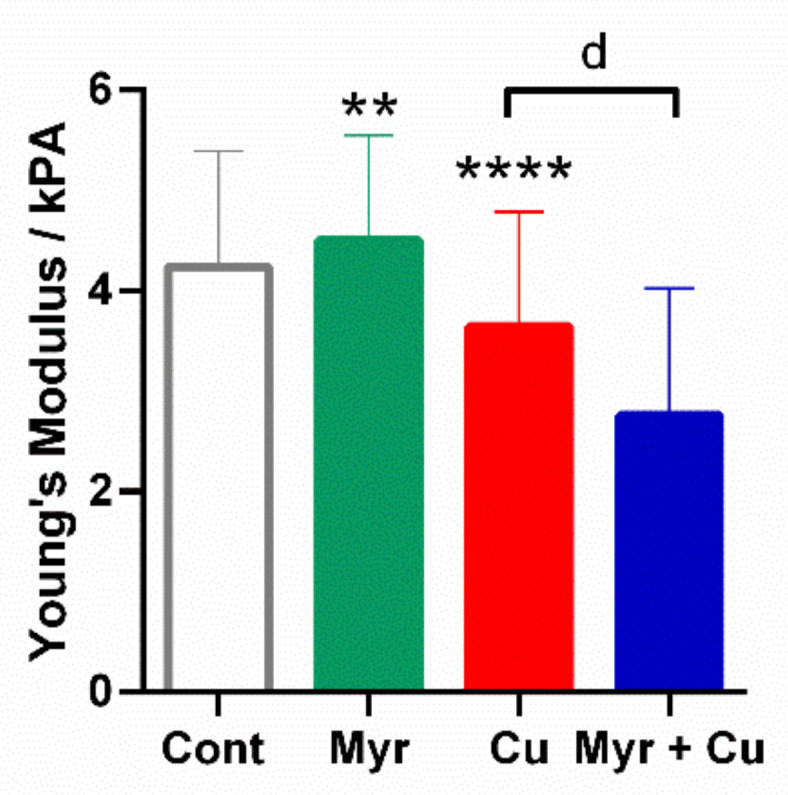
Elasticity analysis of SH-SY5Y cells using AFM. The Young’s modulus values were obtained from force-distance curves (*N*_fc_ = 440 for control cells *N*_cell_ = 8; *N*_fc_ = 480 for each treatment of cells and *N*_cell_ = 8). Results indicate that cell elasticity was increased following its exposure to myricetin only. Copper reduced cellular elasticity, and the effect was more pronounced in the presence of myricetin. Results are represented as mean ± SD. ** *p* < 0.01, **** *p* < 0.0001 vs. control, ^d^ *p* < 0.0001 vs. copper treatment (one-way ANOVA followed by post hoc Tukey’s test).

Since AFM imaging and roughness analysis indicated a significant reorganization of the cytoskeleton and filament structures, we further investigated their nanomechanical properties, i.e., elasticity, as well as a possible correlation between the roughness parameters and nanomechanics of the control and treated cells. Young’s modulus is the crucial elasticity parameter. From 440 force-distance curves (*N*_fc_) measured on control neuroblastoma cells (*N*_cell_ = 8), Young’s moduli values were calculated and plotted on histograms of the measured elasticity (Figure 8). The same procedure was performed in two independent experiments, and Young’s moduli of both experiments were obtained by fitting the Gaussian function as shown in Figure 8A. The elasticity values did not differ significantly, indicating the reproducibility of the obtained data. The same procedures using *N*_fc_ = 480 force-distance curves were repeated for SH-SY5Y cells (*N*_cell_ = 8) treated with myricetin, copper, and with both. Results are presented in Figure 8B–D, summarized in Figure 9 and Table 1. Following treatment with 10 μg/mL of myricetin, the average Young’s modulus was increased from 4.2 ± 1.1 kPa to 4.5 ± 1.1 kPa (Figure 9; *p* = 0.0027, one-way ANOVA followed by Tukey’s multiple comparison test). The results indicate that SH-SY5Y cells treated with myricetin are stiffer than the control group. Following copper treatment, the average Young’s modulus value decreased from 4.2 ± 1.2 kPa to 3.7 ± 1.1 kPa (almost 12%; *p* < 0.0001), indicating that copper-treated cells are softer than control cells and that significant mechanical changes were caused by the addition of copper. When cells were treated with both copper and myricetin, Young’s modulus value was further decreased to 2.8 ± 1.3 kPa (*p* < 0.0001). The observed decrease in elastic properties was more than twofold stronger (36%) in comparison to cells treated only with copper. This indicates that the decrease in Young’s modulus value was mostly determined by myricetin.

## 3. Discussion

Due to their ability to act as strong antioxidants, metal chelators and modulators of redox-dependent signaling pathways, flavonoids are highly appreciated as potential neuroprotective agents against OS-induced neuropathological changes [14,32]. In that regard, we considered that deciphering the role of myricetin in the copper-initiated OS may provide some important insights that will be beneficial from the aspect of novel therapeutic approaches in AD and other neurodegenerative diseases. The neuroprotective effects of myricetin, a flavonoid with strong antioxidative power, have been demonstrated in several models of neuronal oxidative injury [21,22,24]. On the other hand, some reports demonstrated the toxic effects of myricetin that were predominantly attributed to its prooxidative activities. It is well-known that antioxidants may be considered a double-edged sword as they are capable of exerting both antioxidative and prooxidative effects. Antioxidant and prooxidant activities of myricetin and other flavonoids are largely determined by the number and position of hydroxyl groups and their chemical environment, which may include the presence of redox-active transition metals [18,23].

Hence, OS was induced by copper overload since copper dyshomeostasis is an important underlying mechanism in the development and progression of pathological processes in neurodegenerative diseases. Expectedly, exposure to excess copper reduced viability and increased production of ROS in neuroblastoma SH-SY5Y cells, a commonly used model for studying the neuronal response to oxidative injury [33,34,35].

Following entry, divalent copper is reduced to Cu^+^ in the presence of high amounts of intracellular reductants, such as GSH. This further drives redox cycling in the vicinity of hydrogen peroxide via Fenton and Haber–Weiss chemistry and results in the increased formation of hydroxyl radicals and other active oxygen species. Brain mitochondria are highly susceptible to copper toxicity, and impairment of their function may additionally contribute to ROS overproduction during severe injury [35]. Yet another factor participating in ROS increase is the formation of the Cu(I)-[GSH]_2_ complex that is capable of reducing molecular oxygen and yielding a superoxide anion [10]. Accordingly, the cytotoxic effects of copper are largely attributed to the increased generation of ROS and accompanying OS [4,7,8].

When applied together with copper, myricetin at concentrations that were not toxic *per se* exacerbated a copper-induced decrease in viability, and further promoted the generation of ROS, clearly demonstrating its cytotoxic and prooxidative abilities in a copper-containing environment. Hence, our results suggest that the neurotoxic effect of myricetin was affected by the increased generation of ROS. Prooxidative properties of flavonoids are documented throughout literature and are predominantly attributed to their oxidation and redox cycling in the presence of oxygen or transition metals [36]. Redox activities of different flavonoids are highly dependent on the number of phenolic hydroxyl groups that could be easily oxidized. Myricetin possesses six hydroxyl groups and thus has great potential for participating in oxidation and redox cycling. Oxidation of flavonoids can be initiated by divalent copper ions. In that reaction, oxidation of hydroxyl groups of flavonoids yields semiquinone radicals and cuprous (Cu^+^) ions. In the next step, semiquinone radicals reduce molecular oxygen, generating a superoxide anion and oxidized flavonoid in the quinone form, whereas Cu^+^ ions reduce a superoxide anion to hydrogen peroxide and regenerate the Cu^2+^ pool [37]. Generated hydrogen peroxide may then be converted to hydroxyl radicals via the Fenton reaction. This flavonoid-mediated Cu^2+^/Cu^+^ redox cycling is considered to be the major underlying mechanism of prooxidative flavonoid action that drives ROS formation (hydroxyl radical in particular) and results in oxidative damage of important intracellular macromolecules and cell death [5]. Thus, the cytotoxic effect of myricetin in SH-SY5Y cells is likely related to the increased production of ROS and the formation of electrophilic oxidation products that are capable of inducing oxidative damage of various biological targets and jeopardizing cell functioning. Previously, it has been shown that myricetin alone might induce oxidative damage and degradation of nuclear DNA, as well as lipid peroxidation. Both effects were further exacerbated in the presence of divalent copper and assigned to enhanced production of ROS in the Fenton type cycling process [36,37]. Similarly, the complex between myricetin, semiquinone and iron (II) reduced hydrogen peroxide and formed hydroxyl radicals, increasing the amount of ROS produced [23]. Furthermore, myricetin may form stable complexes with Cu^2+^ that are capable of intercalating into DNA and producing ROS via the Fenton reaction, thus causing pronounced DNA damage [18].

Together with the increase in ROS production, exposure to copper depleted the intracellular GSH content. Reduced glutathione (GSH) is one of the major nonenzymatic systems of antioxidative defense due to its free radical and metal scavenging abilities. During the reduction of Cu^2+^ and other toxic oxidizing reagents, GSH is oxidized to GSSG, which depletes the total GSH pool inside the cell [10,38]. Cu^+^ generated in a reaction between GSH and Cu^2+^ may further form a Cu-[GSH]_2_ complex, resulting in additional GSH exhaustion. Besides ROS scavenging activities, GSH represents the largest reservoir of sulfhydryl groups that is also important for its redox regulatory activities. Namely, in OS conditions, GSH participates in protective protein glutathionylation and regulates protein function via thiol/disulfide exchange [34]. Hence, its depletion may lead to the impairment of redox homeostasis and protein functioning, contributing to copper toxicity. Although copper induced a pronounced GSH decrease in SH-SY5Y cells, in the presence of myricetin, GSH levels were not additionally reduced. This could probably be explained by their concentrations. In most cells, the GSH concentration is about 1–10 mM [39], whereas concentrations of copper and myricetin used in the study were 500 µM and 31 µM, respectively. As GSH is around two orders of magnitude in excess over myricetin and only one over copper, it is likely that reactions between copper and GSH have a more prominent effect on the entire GSH pool than reactions between myricetin and GSH. Myricetin is structurally similar to quercetin and is also known as hydroxyquercetin [22]. In P19 neuronal cells, quercetin displayed neurotoxic effects in the presence of excess copper and did not enhance the copper-induced decrease of GSH content when applied at 30 µM concentration, but exacerbated copper effects at 150 µM concentration [5]. Furthermore, the oxidized form of quercetin (quercetin-quinone) is highly reactive toward thiols. It preferentially reacts with GSH, but if GSH concentrations are low, it binds to protein thiols and may cause loss of protein function [40]. Based on the structural homology, similar reactions are expected for myricetin. This means that the copper-induced reduction of the GSH level may promote the toxic effect of myricetin and induce secondary damage simply by promoting the binding of myricetin to cellular proteins and by preventing their proper functioning. In addition to all these possibilities, myricetin inhibits glutathione reductase, an enzyme that regenerates GSH from the GSSG, which may also contribute to the deterioration of antioxidative defense [41].

As revealed in previous studies, various death pathways have been triggered upon copper exposure, depending on the copper concentration used, treatment period and the cellular or animal model under study. Copper overload may result in caspase-dependent and caspase-independent programmed cell death, autophagy and necrosis [19,42,43]. In some studies, exposure to excess copper initiated a classical apoptotic pathway and the activation of executioner caspase-3 [43]. However, in SH-SY5Y cells, copper applied alone did not induce an increase in caspase-3 activity, and this effect was not modified in the presence of myricetin. Similar findings, i.e., a copper-induced increase in ROS production accompanied with the suppression of apoptosis, were observed in zebrafish cells exposed to copper [44] and in malignant cells [45]. In P19 neuronal cells, we also failed to detect caspase activation during mild copper-induced neuronal damage [8,19]. Moreover, the direct inhibitory effect of myricetin on caspase-3 activity has been observed via direct binding to the residues in the active site of the enzyme [24].

Condensed nuclei are a hallmark of both caspase-dependent and caspase-independent cell death. As copper and myricetin induced nuclear condensation without caspase-3/7 activation, it is likely that SH-SY5Y cells predominantly died by caspase-independent death. Based on the myricetin-induced increase in the number of nuclei with chromatin condensation, this mode of death was initiated by copper and then exacerbated in the presence of myricetin. However, some nuclei were also stained with PI, which binds to the DNA of late apoptotic and necrotic cells, suggesting the presence of necrotic processes. In general, necrosis is characterized by rapid loss of cellular membrane potential due to ATP depletion and failure of ion pumps and channels, resulting in swelling and rupturing of the cell membrane [46]. Hence, the most important hallmark of necrotic cells is the loss of membrane integrity. In SH-SY5Y cells exposed only to copper, LDH activity was below the basal levels found in the control and myricetin-treated groups. As previously reported, copper inhibits LDH activity [8,29], preventing a precise assessment of the level of membrane damage following exposure to copper alone. Nevertheless, in cells treated with both copper and myricetin, an increase in LDH activity was obvious, indicating the existence of more pronounced membrane damage following myricetin treatment and death by necrosis.

As already mentioned, copper can react with hydrogen peroxide and yield a particularly dangerous hydroxyl radical in the Fenton reaction. Hence, at cytotoxic concentrations, copper may induce oxidative modifications of DNA bases and DNA strand breaks [47]. Similarly, myricetin may form intercalating complexes with copper that are capable of inducing DNA damage [18]. Poly(ADP-ribose)polymerase-1 (PARP-1) is a nuclear enzyme that catalyzes poly(ADP-ribosyl)ation of nuclear acceptor proteins, including itself. It is activated upon binding to DNA strand breaks and has a critical role in the initiation and modulation of DNA repair pathways [48]. Expression of PARP-1 was elevated in SH-SY5Y cells during copper exposure and was further promoted by myricetin, probably reflecting the more pronounced DNA damage in the presence of the flavonoid. When DNA is extensively damaged, PARP over-activation may result in a bioenergetic collapse, and a specific form of regulated necrotic death termed parthanatos [48,49]. This type of death should be prevented by the PARP inhibitor PJ34 [50,51], but in SH-SY5Y cells, PJ34 exacerbated the cytotoxic effect of myricetin and copper. This suggests that parthanatos is not the underlying mechanism of myricetin-induced cell death and indicates that PARP-1 activation only serves a protective role in our conditions. Yet another type of death was described in organotypic hippocampal slices [52]. Similar to our observations, PARP-1-mediated death of hippocampal neurons was associated with reduced ATP content and a lack of caspase-activation, as well as characterized by the pronounced deterioration of neuronal membrane properties due to the changed expression and function of AMPA receptors [52]. This mode of PARP-1-mediated death has been observed in neurodegenerative diseases, and the expression of AMPA receptors has been confirmed in undifferentiated SH-SY5Y cells [52,53].

Yet another protein with an important role in the cellular response to OS and DNA damage is transcription factor p53. A rapid increase of p53 expression and activity is induced by various oxidizing agents, including copper, and is a contributing factor to death events [8,54]. In the lymphocytes of AD patients, p53 and PARP-1 conferred increased susceptibility to H_2_O_2_-induced OS and death by both apoptosis and necrosis. In that study, a protective role of PARP-1 was also suggested [55]. In SH-SY5Y cells, p53 expression was upregulated following copper exposure but was not additionally upregulated during concomitant treatment with myricetin. Furthermore, pifithrin α, an inhibitor of transcriptional p53 activity, did not modify the outcome of copper and myricetin treatment, suggesting that cell death is not dependent on the transcriptional activation of p53. Necrotic death in OS conditions could be initiated by the opening of the mitochondrial permeability transition pore, which induces massive ion influx, dissipates mitochondrial membrane potential, and blocks ATP production. Opening of this pore is triggered through p53 interactions with cyclophilin D (CypD), a permeability transition pore regulator, and is Bax independent [46]. Perhaps a similar mechanism is operative in SH-SY5Y cells, particularly if considering that changes of Bax were not found in our conditions. In contrast, a myricetin-induced decrease in viability was accompanied by reduced levels of the antiapoptotic protein Bcl-2. Suppression of Bcl-2 expression, together with the increase of the Bax/Bcl-2 ratio, has been observed in various forms of cell death induced by flavonoids [56,57,58], and p53 may promote cell death through transcriptional repression of Bcl-2 [54]. In addition to decreased Bcl-2 expression, both copper and myricetin attenuated the expression of the ΔNp73 isoform. Although expression of the TAp73 isoform was not affected, suppression of ΔNp73 expression disturbed the relative ratio of isoforms, which may be a critical factor in driving cellular response to oxidative injury. Similar to our study, in neuronal death induced by the withdrawal of the nerve growth factor, p53 was upregulated, and ΔNp73 downregulated [59]. Upon DNA damage, ΔNp73 can be rapidly degraded through an unknown mechanism, enabling death events to occur [60]. In contrast, overexpression of ΔNp73 rescues neurons from various apoptotic stimuli by antagonizing the proapoptotic p53 function and through the p53-independent mechanism [61]. Hence, it is likely that DNA damage was implicated in the ΔNp73 degradation, which further contributed to the activation of death events due to the loss of neuroprotective functions of the ΔNp73 isoform.

Exposure to copper and myricetin also had a prominent effect on the expression of nucleoside diphosphate kinase NME1. Its involvement in OS response has been pointed out in previous studies [19,62]. Furthermore, overexpression of NME1 promoted the survival of primary rat cortical neurons during excitotoxicity, hydrogen peroxide-induced OS and oxygen-glucose deprivation [63]. Hence, considering that NME1 may provide protection against OS, a reduction of its expression probably compromises its protective function and can be directly related to the reduced viability of SH-SY5Y cells following exposure to copper and myricetin.

As myricetin may modulate the pattern of phosphorylation of various kinases [24], we investigated if the neurotoxic effect of myricetin could be modified with inhibitors that act along MAPK pathways, which are commonly activated in OS responses [64]. The effect of myricetin on copper-induced death was exacerbated in the presence of the JNK inhibitor SP600125. This indicates that activation of JNK is beneficial for SH-SY5Y cells. Although JNK activation usually results in death, different JNK isoforms may have cell-specific roles and different regulatory effects. The protective role of JNK activation has been documented in various forms of neuronal death [19,65,66]. In fact, the JNK/AP-1 system has been suggested as one of the most important protective mechanisms in neurons as it promotes transcription of antioxidant response proteins [67]. Hence, the observed results suggest that the combination of copper and myricetin has inhibitory effects on JNK activity, probably decreasing the ability of SH-SY5Y cells to cope with OS. ERK1/2 and p38 pathways may participate in p53 stabilization, thus enhancing the proapoptotic functions of p53 [15]. However, SB203580, a specific inhibitor of p38 signaling, did not modulate the effect of myricetin, suggesting a lack of p38 activation. On the other hand, UO126, an ERK1/2 inhibitor, exacerbated the toxic effects of myricetin. This suggests the protective role of ERK signaling but may also indicate that myricetin achieved its neurotoxic effects by inhibiting ERK1/2 and JNK pathways. The inhibitory effects of myricetin on ERK and JNK activities have been confirmed in various experimental settings [21,22].

In the presence of damaged DNA, the catalytic activity of PARP-1 may be stimulated by ERK2 [48]. Hence, the detrimental effects of ERK inhibition on the survival of SH-SY5Y cells may be related to reduced PARP-1 activity and impairment of its function in DNA repair and, ultimately, neurotoxic effects. ERK signaling may also be activated by calcium influx. The involvement of Ca^2+^ ions in copper-induced neuronal death has been confirmed in previous studies [8,68]. It is known that calcium entry via L-type calcium channels may stimulate the ERK pathway in a PKA-dependent manner [69]. Nifedipine, a specific blocker of L-type calcium channels, as well as H-89, the PKA inhibitor, also promoted the toxic effects of myricetin. Thus, reduced viability in the presence of nifedipine and H-89 might also indicate an absence of protective ERK/PARP-1 activation in SH-SY5Y cells. We further investigated if the neurotoxic effects of myricetin could be related to the suppression of Akt activation. Protein kinase B (Akt) is generally considered to be a survival factor that protects neurons from various adverse stimuli, and myricetin is capable of inhibiting Akt activity [70]. However, wortmannin, a covalent inhibitor of PI3K, which is an upstream activator of Akt, did not affect cell survival, suggesting that inhibition of the PI3K/Akt pathway is not involved in the detrimental effects of myricetin.

Among all the tested inhibitors, the neurotoxic effect of myricetin was reversed only by staurosporine, an ATP-competitive kinase inhibitor that binds to many kinases with little selectivity. Thus, it seems that, at least partially, the neurotoxic effects of myricetin were achieved by modulating signaling pathways. Although myricetin may directly inhibit various kinases, inhibition of various pathways may also be a consequence of disturbed redox signaling due to a myricetin-induced ROS increase, which needs to be clarified in further studies.

The cytotoxic effects of myricetin were also investigated by AFM. We aimed to collect high-resolution morphological images and nanomechanical properties under the influence of copper and myricetin in the process of neurodegeneration. AFM represents a powerful tool for the evaluation of cell surface morphology, changes of filamentous cytoskeletal structures, and local mechanical properties at the nanoscale resolution [27,71]. The field of AFM application in neuronal studies is developing and includes the monitoring of structural and mechanical changes induced by treatment with various compounds in OS-conditions [28,72,73,74].

The observed changes in morphology and surface roughness are probably caused by the reorganization of fine filamentous structures leading to the formation of aggregate-like structures. Actin filaments are the main component of the cytoskeleton, and the mechanical properties of cells, including elasticity and surface roughness, are highly determined by their dynamics [71,73]. An increase in surface roughness that is observed after exposure to copper and myricetin suggests degeneration of the actin network [73]. Furthermore, exposure to copper and myricetin also induced a prominent decrease in cell elasticity. It has been shown that disaggregation of actin filaments decreases the average elastic modulus, demonstrating the importance of actin filaments for overall mechanical stability and the ability to resist elastic deformation [71]. A pronounced drop in elastic properties has already been observed in living and fixed cells under exposure to OS. It is considered that a decrease of cell stiffness may result from changes in membrane permeability, overproduction of ROS, disruption of sites where the cytoskeleton connects to the plasma membrane, and the deterioration of the actin network [72,75]. Hence, the AFM results are consistent with cellular assays that indicate a myricetin-related loss of membrane integrity and death by necrosis in the presence of copper. In addition to deciphering the effects of myricetin, force spectroscopy and AFM imaging results provided a better insight into the effects of copper on cell surface topography and nanomechanical properties. Changes that were observed in the neuroblastoma SH-SY5Y cells include depletion of filamentous structures and disruption of the original dynamics of the cytoskeleton, which was particularly enhanced during the myricetin treatment in the presence of copper. A better understanding of the mechanical response can potentially provide an alternative therapeutic window for overcoming the consequences of copper-related neurodegeneration.

## 4. Materials and Methods

### 4.1. Chemicals

4-diamino-2,3-dicyano-1,4-bis[2-aminophenylthio]-butadiene (UO126), 2’,7’-dichlorofluorescin diacetate (DCF-DA), nifedipine, 3-(4,5-dimethylthiazol-2yl)2,5-dyphenyl-2H-tetrazolium bromide (MTT), *N*-[2-*p*-bromocinnamyl(amino)ethyl]-5-isoquinolinesulfonamide (H-89) dihydrochloride hydrate, Hoechst 33342, propidium iodide (PI), staurosporine were purchased from Sigma-Aldrich Chemicals (St. Louis, MO, USA). Wortmannin was purchased from Ascent Scientific (Princeton, NJ, USA). SP600125, SB203580 and PARP inhibitor VIII (PJ34) were from Alfa Esar (Ward Hill, MA, USA). Myricetin was obtained from TCI Chemicals Pvt. Ltd. (Chennai, India), whereas copper sulfate pentahydrate was purchased from Kemika (Zagreb, Croatia). All chemicals used for maintaining SH-SY5Y cells, including culture medium (Dulbecco’s modified Eagle’s medium, DMEM), fetal bovine serum (FBS), trypsin, antibiotics, Na-pyruvate and L-glutamine, were purchased from Sigma-Aldrich (St. Louis, MO, USA) or Gibco (Paisley, U.K.). All other chemicals used were of analytical grade.

### 4.2. Culturing of SH-SY5Y Cells

SH-SY5Y cells are of neuroblastoma origin. They are derived from the bone marrow of a 4-year-old girl. The cells were cultured in a high-glucose DMEM containing 10% heat-inactivated FBS, 2 mM L-glutamine, 100 units/mL penicillin G and 100 µg/mL streptomycin) in a humidified atmosphere of 5% CO_2_ at 37 °C. All experiments were performed with cells between 10 to 20 passages. For assays performed in 96-well tissue culture plates, 20 × 10^3^ cells/well were plated app. 24 h before the treatment, whereas for 6-well format, 650 × 10^3^ cells/well were seeded.

### 4.3. Drug Treatment

For dose–response studies, SH-SY5Y cells were exposed to increasing concentrations of CuSO_4_ (0.1–1.5 mM) and myricetin (1–20 µg/mL) for 24 h. A stock solution of myricetin was prepared in DMSO (10 mM) and diluted with a DMEM medium to final concentrations (1–20 μg/mL). In experiments aimed to investigate the effects of myricetin in copper-induced OS conditions, SH-SY5Y cells were exposed to 0.5 mM CuSO_4_ in the presence of those concentrations of myricetin that did not affect the survival of SH-SY5Y cells when applied alone.

To examine the effects of myricetin on the activation of selected signaling pathways and L-type voltage-gated calcium channel, SH-SY5Y cells were treated with various inhibitors: wortmannin (30 nM)-inhibitor of phosphatidylinositol-3-kinase (PI3K)/Akt pathway, UO126 (1 µM)-inhibitor of extracellular signal-regulated kinases 1/2 (ERK1/2) signaling pathways, SB203580 (10 µM)-inhibitor of p38 signaling, SP6000125 (5 µM)-inhibitor of c-Jun N-terminal kinase (JNK), pifithrin-α (0.5 µM)-p53 inhibitor, PARP inhibitor VIII (PJ34, 5 µM)-inhibitor of poly(ADP-ribose) polymerase (PARP), nifedipine (10 µM)-inhibitor of calcium channels, H-89 (5 µM)-inhibitor of protein kinase A, and staurosporine (1 nM)-a broad-spectrum kinase inhibitor. All inhibitors were present 60 min prior and during the exposure to 0.5 mM copper and myricetin. The concentrations of inhibitors were chosen based on our preliminary experiments. For each inhibitor, we applied the highest concentration that did not modify viability when applied alone.

### 4.4. Assessment of Cell Viability

#### 4.4.1. MTT Assay

Cell survival was estimated based on the ability of viable cells to cleave MTT to an insoluble formazan product due to the activity of mitochondrial dehydrogenases. At the end of the incubation period, 40 μL of MTT solution prepared in DMEM medium (final concentration 0.5 mg/mL) was added to each well and incubated for 3 h at 37 °C. Precipitated formazan was dissolved by adding 160 μL of dimethyl sulfoxide (DMSO). The absorbance of each well was recorded by an automatic microplate reader at 570 nm. After blank subtraction from all absorbance readings, cell viability was expressed as the percentage of absorbance of myricetin- and/or copper-treated cells relative to that of the untreated control group.

#### 4.4.2. Trypan Exclusion Assay

Survival of SH-SY5Y cells was also determined by the trypan blue exclusion assay. Healthy cells can exclude the dye from their cytoplasm and thus appear bright under the microscope, while cells with compromised membrane integrity lose this ability and appear blue. Following treatment in 6-well culture plates, the culture medium with floating cells was collected into a centrifuge tube (15 mL). The attached cells were trypsinized for 10 min, resuspended in DMEM and pooled with their corresponding medium. Cells were centrifuged at 250 g for 5 min; pellets were resuspended in 250 μL of DMEM medium and incubated for 5 min in the presence of 0.4% trypan blue. The ratio of trypan blue-stained nuclei over the total number of cells was used to determine the percentage of cell death. At least 500 neurons were counted in each group per experiment.

#### 4.4.3. Determination of ATP Level

Following exposure to copper and/or myricetin, cell survival was also determined by using the CellTiter-Glo 2.0 assay (Promega) according to the manufacturer’s instructions. This assay measures ATP generated by metabolically active cells based on an ATP-dependent luciferase reaction. Briefly, at the end of the treatment period, 100 µL of CellTiter-Glo reagent was added to each well. The content of the plate was mixed on an orbital shaker for 2 min to induce cell lysis. After a 10-min incubation at RT, luminescence was recorded in a luminometer with an integration time of 0.8 s per well (Fluoroskan Ascent FL, Thermo Scientific).

### 4.5. Measurement of Lactate Dehydrogenase (LDH) Release from SH-sY5Y Cells Exposed to Copper and Myricetin

The effect of copper and myricetin treatment on the plasma membrane integrity was assessed by a fluorometric assay (CytoTox-ONE™ homogeneous membrane integrity assay, Promega, WI, USA). This assay is based on the principle that cell death during necrosis results from the breakdown of the plasma membrane, thus enabling the release of the cytoplasmic enzyme LDH into the culturing medium [76].

Following a 24 h treatment, aliquots of the culture (100 μL) were taken from the 6-well tissue culture plates. The culture medium was then combined with the 100 μL of assay mixture (containing lactate, NAD^+^, and resazurin as substrates in the presence of diaphorase) and incubated for 60 min at 37 °C. Generation of the fluorescent product resorufin is proportional to the amount of LDH released. Fluorescence was determined by Fluoroskan Ascent FL luminometer (Thermo Scientific) by using an excitation wavelength of 530 nm and an emission wavelength of 590 nm.

### 4.6. Measurement of Intracellular ROS Accumulation

Intracellular production of ROS was detected by using the cell-permeable substrate DCF-DA. DCF-DA diffuses into the cells where it is hydrolyzed to non-fluorescent 2′,7′-dichlorofluorescin by intracellular esterases. The generated 2′,7′-dichlorofluorescin then reacts with intracellular ROS and forms the highly fluorescent compound dichlorofluorescein (DCF). The method is considered to be a reliable indicator for total ROS production and is efficient in evaluating the potency of various pro-oxidants [77].

Briefly, at the end of the treatment period, SH-SY5Y cells were incubated with 100 μM DCF-DA in PBS for 30 min in darkness and then incubated for an additional 1 h in PBS. The emitted fluorescence was determined using a Fluoroskan Ascent FL luminometer at an excitation wavelength of 485 nm and an emission wavelength of 538 nm. The results are expressed as the percentage of the fluorescence intensity recorded in the control group.

### 4.7. Determination of Reduced Glutathione (GSH) Levels

Glutathione is one of the most abundant nonenzymatic antioxidants found in cells. Most of the intracellular glutathione is present in the reduced form (GSH), whereas only a small part is oxidized and found as a dimer in which two sulfhydryl groups are connected by a disulfide bond (GSSG).

Changes in GSH levels were monitored with the luminescence-based GSH-Glo glutathione assay (Promega, Madison, WI, USA) according to the manufacturer’s instructions. Essentially, the assay couples two chemical reactions. The first reaction is catalyzed by glutathione-S-transferase and converts a GSH probe, luciferin-NT, into luciferin. In the second reaction, catalyzed by firefly luciferase, the amount of luciferin produced is detected as a luminescent signal. The overall intensity of the signal is proportional to the GSH amount present in each sample. At the end of exposure to copper and myricetin, the medium was removed, and 100 μL of the GSH-Glo reagent was added to each well. The plates were incubated for 30 min at RT in the dark. Thereafter, 100 μL of the luciferin detection reagent was added, mixed briefly, and incubated for a further 15 min. Emitted luminescence was quantified by using a Fluoroskan Ascent FL luminometer (Thermo Fisher Scientific, Waltham, MA, USA).

### 4.8. Determination of Caspase -3/7 Activity

Caspase-3/7 activity was determined by using the caspase-Glo 3/7 assay system (Promega, Madison, WI, USA). These two caspases are key effector enzymes in caspase-dependent apoptosis and are commonly activated in ROS-triggered neuronal death. The assay provides a luminogenic substrate with the tetrapeptide sequence DEVD.

To perform the assay, tissue culture plates containing cells in 80 μL of DMEM medium were removed from the incubator and allowed to equilibrate to room temperature. Then 80 μL of caspase-Glo 3/7 reagent was added to each well and mixed with a plate shaker at 500 rpm for 30 s. This resulted in cell lysis, caspase cleavage of the proluciferin DEVD substrate, and release of aminoluciferin, a substrate for luciferase. Ultimately, the luminescence generated in the reactions is proportional to the intensity of caspase activity in each sample. The plates were incubated at room temperature for 90 min. The luminescent signals were recorded by a Fluoroskan Ascent FL luminometer (Thermo Fisher Scientific, Waltham, MA, USA).

### 4.9. Nuclear Staining with Hoechst 33342 and Propidium Iodide (PI)

To better characterize the type of death induced by copper and myricetin exposure, we looked for changes in chromatin morphology. Cells were stained with 1 µg/mL Hoechst 33342 and 1 µg/mL PI for 5 min in the dark, directly in the tissue culture dish in the DMEM medium. The photographs were taken with the EVOS Floid cell imaging Station (Thermo Fisher Scientific, Waltham, MA, USA). Quantitative analysis was performed by counting more than 500 cells from photographs obtained in three separate experiments.

### 4.10. Western Blot Analysis of PARP-1, p53, p73, Bcl-2, Bax and NME1/2 Protein Expression

Following treatment, the whole-cell lysates were prepared by scraping cells into PBS with protease inhibitors (Complete, Mini, EDTA-free protease inhibitor cocktail tablets; Roche, Indianapolis, IN, USA) and sonicated (1 mm probe, 2 × 15 s). The protein concentration was determined using the Pierce BCA protein assay kit (Thermo Fisher Scientific, Waltham, MA, USA). Extracted proteins (50 μg) were resolved using SDS–PAGE and analyzed by immunoblotting on nitrocellulose membranes (Whatman, GE Healthcare, Life Sciences, Berlin, Germany). Nonspecific binding was blocked by 5% nonfat milk in Tris-buffered saline containing 0.05% Tween-20 (TBST) for 30 min at RT. Immunoblots were incubated overnight with primary antibody and then for 1 h at RT by appropriate secondary antibody. Blots were incubated with primary anti-PARP-1 antibody (F-2: sc-8007; Santa Cruz Biotechnology, 1:1000), anti-p53α antibody (TSRα kindly provided by Prof. J-C. Bourdon, Dundee, United Kingdom; 1:2000), anti-p73 antibody (ab40658, Abcam, 1:3000), anti-Bcl-2 antibody (C-2: sc-7382; Santa Cruz Biotechnology, 1:1000), anti-Bax antibody (B-9: sc-7480; Santa Cruz Biotechnology; 1:500) and anti-NME1/NME2 antibody (kindly provided by I. Lascu, Bordeaux, France and S. Volarević, Rijeka, Croatia; 1:3000). β-actin (7D2C10, 60008-1-Ig, Proteintech; 1:10,000) was used for normalization. Immunoreactive bands were visualized by chemiluminescence detection (Western Lightning Plus-ECL Enhanced Chemiluminescence Substrate, PerkinElmer, Waltham, MA, USA) and quantified by using ImageJ NIH software after detection with Alliance 4.7 (UVItec Cambridge, London, UK).

### 4.11. AFM Measurements

AFM cell imaging, surface roughness and force measurements were performed using a multimode scanning probe microscope with a Nanoscope IIIa controller (Bruker, Billerica, MA, USA) equipped with a vertical engagement (JV) 125 mm scanner. An optical camera was coupled to the AFM system to locate the position of the AFM probe and the sample. The fluid cell (Bruker, Billerica, MA, USA) was washed with ultra-pure water and ethanol before each experiment. All the measurements were obtained with the same triangular tip type (MSNL, Bruker) of spring constant (0.07 N m^−1^) and frequency (22 kHz). During imaging and force measurements, the temperature was tuned to 37 °C with a temperature controller stage (Digital Instruments, high-temperature heater controller, with resolution 0.1 °C and accuracy 3%). During scanning, both trace and retrace images were recorded and compared for accuracy. Furthermore, morphological properties of the distinct cell regions, i.e., the central part of the cell and seven locations around the central part of the cell randomly chosen (5 μm × 5 μm) were acquired, as well as a detailed image of 2 μm × 2 μm at the optimized imaging conditions to avoid any possible influence of the applied force on imaging and to provide more accurate measurements of the height and structure of the individual subcellular regions. In addition, in order to avoid and reduce the aforementioned effects of time-consuming measurements and imaging, the location around the central part of the cell was randomly chosen. The spring constant of the cantilever was calibrated using the reference cantilever method [78] with a reference cantilever after piezo sensitivity (Vm^−1^) was determined by measuring it at high voltages and after several minutes of performing force plots on a clean glass surface to avoid hysteresis. During a force spectroscopy experiment, the tip consecutively approached and left the cell surface (speed of 2 μm s^−1^). Typically, force curves were taken at around 450 points for each sample at the highest part of the cells at several different points (randomly chosen) across a cell surface to average slight deviations in cell surface and take representative measurements. Extraction of the elasticity from the force measurements has been performed as previously described [79].

The glass plate with the sample cells within a Petri dish was sealed on the standard sample holder using two-component rubber glue. Before imaging, cells were examined with an optical camera, DMEM media was added to the fluid cell, and cells of interest were selected. For each group, we analyzed several cells (*N*_cell_ = 8) from two independent experiments. Processing and analysis of images were carried out using the NanoScopeTM software (Digital Instruments, Version V614r1 and V531r1). All images are presented as raw data except for the first-order two-dimensional flattening.

### 4.12. Statistical Analysis

Statistical analysis of the data was performed by using GraphPad Prism software (San Diego, CA, USA). Comparisons between group means were evaluated by one-way ANOVA, and when statistically significant, post hoc analysis with Dunnett’s or Tukey’s multiple comparison test followed. The normality of the data distribution was verified by D’Agostino–Pearson’s test. The statistical significance level was set at α = 0.05.

## 5. Conclusions

In conclusion, we found that myricetin, a flavonoid with a well-described antioxidant activity, exacerbated the toxic effects of copper and reduced cell viability. It seems that the major driver of the neurotoxic effect of myricetin is an increased production of ROS through autooxidation and redox cycling, which leads to apoptotic and necrotic death, accompanied by the modification of intracellular signaling pathways and changes in the expression of proteins involved in the OS response and initiation of death cascades. A nanomechanical approach also revealed the degenerative behavior of neuroblastoma SH-SY5Y cells. Here, we demonstrated that AFM presents a superior and effective tool for monitoring cellular integrity (e.g., morphology, roughness and elasticity) and for quantifying mechanical properties as biophysical markers for screening neurodegenerative changes.

## Figures and Tables

**Table 1:** Morphological and nanomechanical characteristics of neuroblastoma SH-SY5Y cells.

	Treatment			
	Control Cells	Myricetin (10 μg/mL)	Cu^2+^ (0.5 mM CuSO_4_)	Myricetin (10 μg/mL) + Cu^2+^ (0.5 mM CuSO_4_)
*Z* range (*N*_cell_ = 8)	353 ± 227	389 ± 190	514 ± 123	540 ± 196
*R*_q_/nm (*N*_cell_ = 8)	47 ± 27	44 ± 34	66 ± 27	89 ± 45
*R*_a_/nm (*N*_cell_ = 8)	33 ± 16	35 ± 28	54 ± 23	71 ± 35
*E* (*N*_cel L_ = 8)/kPa	4.2 ± 1.1	4.5 ± 1.1	3.7 ± 1.1	2.7 ± 1.3

## Data Availability

The data presented in this study is available in this article.
